# The effects of soymilk plus probiotics supplementation on cardiovascular risk factors in patients with type 2 diabetes mellitus: a randomized clinical trial

**DOI:** 10.1186/s12902-023-01290-w

**Published:** 2023-02-10

**Authors:** Azimeh Hasanpour, Siavash Babajafari, Seyed Mohammad Mazloomi, Mesbah Shams

**Affiliations:** 1 Department of Nutrition, School and Research Center of Nutrition and Food Sciences, Shiraz University of Medical Sciences, Shiraz, Iran; 2 Department of Food Hygiene and Quality Control, School and Research Center of Nutrition and Food Sciences, Shiraz University of Medical Sciences, Shiraz, Iran; 3 Department of Internal Medicine, Namazi Hospital, Shiraz University of Medical Sciences, Shiraz, Iran

**Keywords:** Soymilk, Probiotics, Type 2 diabetes mellitus, T2DM, Cardiovascular risk factors

## Abstract

**Objective:**

Type 2 diabetes mellitus (T2DM) is associated with an increased risk of cardiovascular diseases. This study aimed to assess the effects of soymilk plus probiotics co-administration on cardiovascular risk factors in T2DM patients.

**Methods:**

One hundred patients with T2DM (aged 40–75 years old) were randomly assigned into 4 groups (soymilk + probiotics supplement, soymilk + placebo, conventional milk + placebo, and probiotics supplement) for 6 weeks. Standard protocols were followed for the collection of fasting blood samples, dietary intakes, and anthropometric measurements.

**Results:**

It was shown that soymilk + probiotics consumption significantly decreased diastolic blood pressure (DBP) (*p* = 0.001), triglycerides (TG) (*P* < 0.001), total cholesterol (TC) (*p* < 0.01), and insulin (*P* < 0.003) levels and significantly increased high-density lipoprotein cholesterol (HDL-C) (*P* = 0.002) levels. Soymilk + placebo administration significantly decreased DBP (*p* = 0.01), insulin (*p* = 0.006), and TG (*p* = 0.001) levels and significantly increased HDL-C (*p* = 0.03) levels. A significant decrease in insulin (*p* = 0.003) and systolic blood pressure (SBP) (*p* = 0.01) levels and an increase in HDL-C (*p* = 0.04) levels were observed after supplementation with probiotics. Findings from between-group comparisons showed a significant decrease in SBP levels in the probiotics supplement group compared to conventional milk group (*p* < 0.05).

**Conclusion:**

Soymilk and probiotics consumption might improve some cardiovascular risk factors in patients with T2DM. However, possible synergic effects while consumption of soymilk plus probiotics supplement didn’t show in this study which warranted further research.

## Introduction

Globally, the number of people with type 2 diabetes mellitus (T2DM) is increasing rapidly [1]. There is an urgent need to determine the causative factors and to develop novel treatments as a result of their increasing prevalence [2]. Recently, interest has been drawn toward the role of the intestinal microbiota as a potential novel contributor to this disease [3]. The intestinal microbiota is believed to impact on energy balance by aiding in the metabolism of nutrients from the diet and contributing to the storage and expenditure of energy [4]. There is evidence that probiotics (live microorganisms or microbial mixtures) can modify the composition of gut bacteria and benefit their hosts [5]. It has been observed that different probiotic strains had favorable effects on the indicators of T2DM in animal studies [6–9]. However, in a systematic review and meta-analysis, probiotics supplementation marginally reduced the levels of fasting blood sugar (FBS) and fasting insulin, but not the HbA1c or the homeostasis model assessment of insulin resistance (HOMA-IR) [10]. It was also shown to improve some metabolic outcomes in patients with T2DM statistically, but not clinically, according to another meta-analysis [11].

Soy and soy products have been shown to have protective effects against a variety of outcomes, including cardiovascular disease, kidney disease, bone loss, cancer, and menopausal symptoms [12–17]. In a meta-analysis, soy products and soy constituents (soy protein and soy isoflavones) were associated with a lower risk of developing T2DM [18]. However, a meta-analysis study reported that consumption of soy products had significant effects in the reduction of some but not all cardiovascular risk factors in patients with T2DM [19]. Among soy products, soymilk is rarely used due to its bad taste [20]. In recent years, however, soymilk has been shown to have positive effects on diabetic variables [21–24]. Additionally, studies have shown that soymilk has a higher absorption rate of isoflavones than other soy products [25–27]. Moreover, it has been suggested that the consumption of probiotics with soy products could improve metabolic profiles [28]. Nevertheless, previous studies have examined the effects of soymilk fermented with probiotics on cardiovascular risk factors in patients with T2DM [21–24, 29], and as far as we know no reports are available indicating the effects of soymilk plus probiotics supplements on T2DM parameters. In addition, it seems there is a synergistic effect between soy products and probiotics bacteria [23]. Hence, we hypothesized that probiotic intake might alter phytoestrogen metabolism and enhance the effects of soymilk by altering the gut microbiota. Therefore, this study sought to determine whether co-administration of soymilk and probiotics have additive effects on anthropometric measurements, plasma lipid parameters, and glycemic indices in T2DM patients.

## Methods

### Design and participants

This study included patients with T2DM aged 40–75 years old who were referred to Motahari hospital in Shiraz city, Iran. The patients were considered diabetic if they had an FBS level of 126 mg/dL or higher or were taking glucose-lowering agents or insulin injections. They were not allowed to participate if they were pregnant, lactating, using hormone replacement therapy, drinking alcohol, or smoking cigarettes. Additionally, we did not include people with breast cancer, allergic to soy milk or cow's milk, and people who took antibiotics three weeks before or during the study period. A patient who consumed food or products containing probiotic bacteria three weeks before or during the study period, who changed the dosage of oral antidiabetic drugs, insulin therapy or lipid-lowering drugs, or who did not follow the recommended diet, was excluded from study.

The sample size for this study was calculated based on the results of a previously published study (n = $\frac{2{\delta }^{2}{({Z}_{1-\frac{\alpha }{2}}+{Z}_{1-\beta })}^{2}}{{(d)}^{2}}$) [30] where α = 0.05, β = 0.2, d = 0.3, standard deviation (SD)1 = 12.6, and SD2 = 20.61. Accordingly, 88 patients would be required to complete the study. But to compensate for possible exclusions and losses to follow-up, we recruited 100 diabetics. The study was approved by the Research Council and Ethics Committee of Shiraz University of Medical Sciences (IR.SUMS.REC.1394.62). In addition, this study was registered in the Iranian Registry of Clinical Trials (identification number: IRCT2015080423495N1) (22/01/2016).

### Study procedure

This double-blind randomized clinical trial was conducted on T2DM patients who were referred to Motahari hospital in Shiraz city, Iran. In the beginning, a participant's disease was diagnosed with the help of an endocrinologist. Then, the study procedure was explained to the participants and informed written consent was obtained from all. After that, the participants entered a two-weeks run-in period. During the run-in time, they had to stop taking any soy product, probiotics food or probiotics supplements. After the run-in time, the patients were randomly allocated to 4 groups using the software as follows; group 1; received soymilk (240 cc) + probiotics capsule, group 2; received soymilk + placebo capsule, group 3; received conventional milk (240 cc) + placebo capsule, and group 4; received probiotics capsule. Also, each participant's energy intake was calculated using the estimated energy requirement formula, and the weight stability diet was written for all of them. Energy distribution for all subjects consisted of 55% carbohydrate, 18% protein, and 27% fat. In group 4, the energy that groups 1 and 2 received through soymilk was compensated through other food groups. All patients were asked to maintain their usual diet and physical activity throughout the study period.

### Interventions preparation and compliance to study protocol

Soymilk and cow milk were respectively produced by Soya Sun Company and Mihan-Dairy Company in Iran. The amount of soymilk that should be consumed was determined based on a study conducted in Iran [30]. In that study, one glass of this product improved some factors in patients with T2DM patients without causing any significant adverse effects. The probiotics capsules (FamiLact®) were provided by the bio-fermentation company of the Pharmaceutical Development Center of the. The FamiLact ® is a synbiotic formulation and contains beneficial bacterial strains (*Lactobacillus rhamnosus*, *Lactobacillus casei, Lactobacillus bulgaricus, Lactobacillus acidophilus, Bifidobacterium breve, Bifidobacterium longum*, and *Streptococcus thermophilus*) plus fructooligosaccharides as prebiotic. The amount and type of probiotics bacteria were also determined based on a study conducted by Moroti et al. [31]. f Placebo capsules contained starch and were quite similar to placebo probiotics capsules. The probiotics and placebo capsules were identically packed and coded by the producer to guarantee to the blind. The participants were asked to eat probiotics capsule (one capsule/day) half an hour before soymilk consumption. The patients were visited every two weeks and were evaluated for their adherence to the prescribed diet and soymilk or cow’s milk intake. Compliance with the interventions and physical activity was monitored by the use of 24 h diet recall and physical activity questionnaire every 2 weeks. We found no differences between the prescribed amount and the reported dietary intake in 4 groups throughout the study.

### Measurements

Anthropometric measurements, including height and body weight, were assessed in all patients at baseline and at the end of the study period. Standing and shoeless heights and body weight were measured while participants were minimally clothed with no shoes using digital scales. Body mass index (BMI) was calculated as body weight (kg)/height^2^ (m). Blood pressure was measured three times after the participants sat for 15 min and the mean of the three times measurement was reported. Blood samples were collected after 12 h of fasting overnight. We measured FBS and lipid profiles by an enzymatic colorimetric method using standard kits (Pars Azmoon co, Iran) by auto-analyzer BT1500. Fasting insulin level was measured using monobind insulin ELISA kit (made in USA) using a microplate reader machine (Stat Fax, USA). The HOMA-IR was calculated based on the previous research [32].

### Statistical analysis

Statistical tests were conducted using SPSS 22 and *P* < 0.05 were considered statistically significant. Kolmogorov–Smirnov test was used to test the normality of the distribution of variables. One-way analysis of variance (ANOVA) and chi-square tests were used for the comparison of quantitative and qualitative variables between the groups, respectively. An analysis of covariance (ANCOVA) and a Bonferroni post hoc test were used for comparisons between the groups post-intervention after adjusting for baseline values plus other confounders such as age, sex, BMI, and physical activity. Paired sample t-test was used to compare the changes in quantitative variables in each group before and after the study.

## Results

**Fig. 1:**
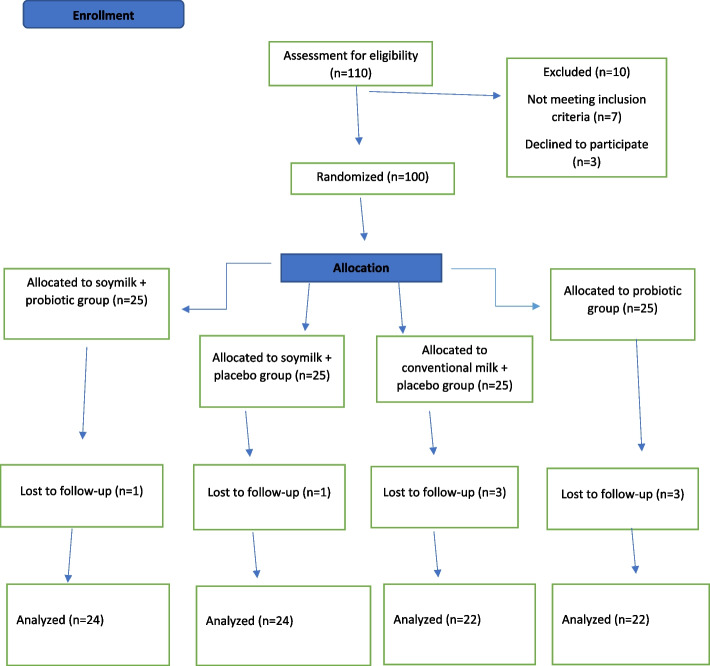
Flowchart of the study

In this study, a total of 100 patients with T2DM, 40–75 years old, were enrolled and randomized to receive one of the 4 dietary regimens for 6 weeks. During the study period, two patients withdrew due to gastrointestinal problems following soymilk consumption. Also, three patients were excluded due to no consumption of more than 3 capsules of probiotics. Moreover, three patients were omitted from the final analyses because of incomplete dietary records. Accordingly, 92 participants (soymilk + probiotics = 24, soymilk + placebo = 24, conventional milk + placebo = 22, and probiotics capsule = 22) completed this study (A flow chart depicting the study design is shown in Fig. 1).

At baseline, there were no significant differences between the 4 groups in terms of sex, demographic status (education and job), anthropometric measurements, and blood pressure parameters (*p* ˃0.05). However, physical activity was significantly different between the groups (*p* < 0.03) (Table 1). The results showed no significant differences between the 4 groups in terms of age, BMI, systolic and diastolic blood pressure, and physical activity levels during and at the end of the study (*p*˃0.05) (data did not show).

**Table 1:** General characteristics of the study participants in each group at baseline

Variables	Soymilk + probiotic supplement (*n* = 25)	Soymilk + placebo (*n* = 25)	Milk + placebo (*n* = 25)	Probiotic supplement (*n* = 25)	^*^P-value
**Age (y)**	51.16 ± 7.16	54.24 ± 6.58	52.06 ± 11.42	54.40 ± 8.72	0.52
**Weight (kg)**	76.50 ± 16.40	75.92 ± 12.02	70.42 ± 14.10	73.88 ± 14.40	0.44
**Body mass index (kg/m2)**	29.06 ± 5.67	28.55 ± 4.46	26.98 ± 5.04	28.05 ± 5.46	0.54
**Systolic blood pressure (mmHg)**	126.45 ± 20.87	129.60 ± 17.07	132.00 ± 18.20	127.60 ± 12.91	0.70
**Diastolic blood pressure (mmHg)**	80.41 ± 9.88	81.40 ± 9.41	81.40 ± 8.48	83.40 ± 7.72	0.69
**Physical activity (Met/hour/day)**	28.17 ± 2.11	28.64 ± 2.30	27.38 ± 2.10	26.99 ± 1.91	0.03
**Gender**					0.12
Female	5 (20)	7 (28)	5 (20)	10 (40)	
Male	20 (80)	18 (72)	20 (80)	15 (60)	
**Occupation**					0.29
Household/retired	6 (24)	9 (36)	7 (28)	12 (48)	
Employed	19 (76)	16 (64)	18 (72)	13 (52)	
**Education**					0.09
Illiterate	20 (80)	23 (92)	18 (72)	23 (92)	
literate	5 (20)	2 (8)	7 (28)	2 (8)	

Data are presented as mean ± SD for quantitative and number (%) for qualitative variables

^*^One-way analysis of variance (ANOVA) and chi-square tests were used for comparison of quantitative and qualitative variables between the groups

*P* < 0.05 was considered statistically significant

In Table 2, dietary intakes from each intervention group are shown at the beginning, during, and after the study. Accordingly, no significant differences were found between the groups regarding dietary intakes in 3 time periods (*p*˃0.05). Also, paired sample t-tests did not show any significant difference between the means of dietary intakes in each group before and after the interventions (*p* < 0.05).

**Table 2:** Dietary intakes of the study participants at beginning, during, and at the end of the study

Variables		Soymilk + probiotic supplement	Soymilk + placebo	Milk + placebo	Probiotic supplement	^*^P-value
**Energy (kcal/day)**	Week 0	1652.17 ± 782.72	1393.49 ± 408.35	1388.77 ± 546.22	1586.25 ± 860.03	0.44
	Week 3	1.68 ± 2.03	0.99 ± 0.23	1.68 ± 2.03	0.99 ± 0.23	0.85
	Week 6	1.68 ± 2.03	0.99 ± 0.23	1.68 ± 2.03	0.99 ± 0.23	0.98
	^**^P-value	0.53	0.22	0.10	0.83	
**Protein (gr/day)**	Week 0	56.47 ± 35.57	47.04 ± 16.42	43.05 ± 24.05	46.79 ± 21.80	0.33
	Week 3	53.47 ± 23.14	50.72 ± 19.28	54.06 ± 34.17	51.90 ± 15.10	0.97
	Week 6	51.20 ± 19.26	52.63 ± 21.90	52.18 ± 27.62	48.20 ± 17.94	0.91
	^**^P-value	0.34	0.24	0.12	0.73	
**Carbohydrate (gr/day)**	Week 0	248.70 ± 114.08	214.09 ± 66.53	213.76 ± 76.65	247.16 ± 93.04	0.36
	Week 3	236.53 ± 83.61	234.45 ± 70.90	252.07 ± 103.35	266.33 ± 73.96	0.56
	Week 6	237.54 ± 90.84	239.68 ± 110.42	245.08 ± 70.18	260.80 ± 73.08	0.19
	^**^P-value	0.57	0.20	0.06	0.53	
**Fat (gr/day)**	Week 0	50.27 ± 32.66	40.82 ± 17.09	42.60 ± 24.28	48.28 ± 70.92	0.85
	Week 3	46.88 ± 21.30	41.87 ± 16.94	49.65 ± 29.08	36.92 ± 14.53	0.21
	Week 6	49.18 ± 19.91	41.52 ± 16.94	45.58 ± 23.21	37.21 ± 16.31	0.81
	^**^P-value	0.83	0.85	0.33	0.47	
**SFA (gr/day)**	Week 0	14.63 ± 11.65	11.28 ± 7.16	11.60 ± 6.84	11.67 ± 8.75	0.54
	Week 3	14.40 ± 8.69	13.22 ± 6.66	15.15 ± 11.75	10.79 ± 5.49	0.35
	Week 6	14.14 ± 7.73	12.09 ± 6.29	13.83 ± 9.44	10.44 ± 4.77	0.30
	^**^P-value	0.80	0.65	0.07	0.55	
**MUFA (gr/day)**	Week 0	14.57 ± 11.93	12.05 ± 7.71	12.52 ± 8.44	12.47 ± 14.30	0.86
	Week 3	13.84 ± 7.20	12.35 ± 6.81	14.89 ± 8.90	10.86 ± 5.82	0.38
	Week 6	14.52 ± 6.16	12.55 ± 7.04	13.27 ± 7.70	11.09 ± 6.89	0.41
	^**^P-value	0.98	0.73	0.48	0.66	
**PUFA (gr/day)**	Week 0	14.78 ± 11.85	11.66 ± 4.38	14.24 ± 9.18	19.72 ± 46.32	0.74
	Week 3	13.50 ± 7.69	10.48 ± 3.91	14.21 ± 8.85	10.25 ± 3.92	0.10
	Week 6	14.79 ± 9.11	11.90 ± 5.73	12.48 ± 7.82	10.85 ± 5.25	0.30
	^**^P-value	0.99	0.83	0.19	0.38	
**Dietary fiber (gr/day)**	Week 0	11.07 ± 4.97	13.49 ± 5.62	12.22 ± 5.22	14.31 ± 5.44	0.21
	Week 3	13.16 ± 5.68	11.16 ± 4.11	11.44 ± 4.53	14.29 ± 5.01	0.12
	Week 6	14.48 ± 7.10	11.39 ± 4.99	10.97 ± 4.13	13.18 ± 6.08	0.14
	^**^P-value	0.16	0.21	0.67	0.08	
**Cholesterol (mg/day)**	Week 0	170.32 ± 158.10	129.81 ± 106.92	161.71 ± 251.43	109.02 ± 108.46	0.58
	Week 3	161.04 ± 105.02	131.75 ± 87.91	209.85 ± 185.70	132.70 ± 80.00	0.13
	Week 6	194.71 ± 129.01	156.12 ± 111.00	156.11 ± 135.31	157.04 ± 130.95	0.66
	^**P^-value	0.48	0.65	0.89	0.08	

Abbreviations: *SFA* saturated fatty acid, *MUFA* monounsaturated fatty acid, *PUFA* polyunsaturated fatty acid

Values are expressed as means ± SD. *P* < 0.05 was considered as statistically significant

^*^One-way analysis of covariance (ANOVA) test was used for comparisons of quantitative variables between the groups

^**^Paired sample t-test was used for comparisons between the means before and after the interventions

*P* < 0.05 was considered statistically significant

Changes in the BMI, SBP, DBP, and biochemical variables from baseline to the end of the study are summarized in Table 3. Following soymilk + probiotics supplementation, DBP (*p* = 0.001), TC (*p* = 0.01), TG (*p* < 0.001), and insulin (*p* = 0.003) levels were significantly decreased, but HDL-C (*p* = 0.002) levels significantly increased. It was also found that supplementation with soymilk + placebo significantly decreased DBP (*p* = 0.01), TG (*p* = 0.001), and insulin (*p* = 0.006) levels, while significantly increasing HDL-C (*p* = 0.03) levels. The levels of HDL-C (*p* = 0.04) significantly increased and the levels of insulin (*p* = 0.003) and SBP (*p* = 0.01) significantly decreased when the participants received probiotics supplements.

**Table 3:** The effects of supplementation with soymilk and probiotic on anthropometric measures and biochemical markers of patients with T2D

Variables		Group 1: Soymilk + probiotic supplement (*n* = 24)	Group 2: Soymilk + placebo (*n* = 24)	Group 3: Milk + placebo (*n* = 22)	Group 4: Probiotic supplement (*n* = 22)	^*^P-value
**BMI (kg/m**^**2**^**)**	Before	29.06 ± 5.67	28.55 ± 4.46	26.98 ± 5.04	28.26 ± 5.86	0.88
	After	28.16 ± 7.99	28.46 ± 4.70	27.73 ± 4.83	28.02 ± 5.86	
	Mean change	0.10 ± 0.35	0.01 ± 0.33	0.10 ± 0.42	0.10 ± 0.34	
	^**^P-value	0.36	0.75	0.21	0.36	
**SBP (mmHg)**	Before	126.45 ± 20.87	129.60 ± 17.07	132.00 ± 18.20	127.60 ± 12.91	0.01
	After	119.79 ± 12.02	124.04 ± 14.79	129.09 ± 17.08	119.32 ± 33.84^a^	
	Mean change	-3.15 ± 12.15	-3.86 ± 11.67	-3.52 ± 11.79	-6.40 ± 9.54	
	^**^P-value	0.07	0.06	0.29	0.01	
**DBP (mmHg)**	Before	80.41 ± 9.88	81.40 ± 9.41	81.40 ± 8.48	83.40 ± 7.73	0.02
	After	72.70 ± 10.73	73.69 ± 12.17	79.54 ± 9.50	79.54 ± 4.85	
	Mean change	-6.95 ± 10.55	-5.43 ± 7.49	-3.52 ± 10.48	-6.02 ± 12.40	
	^**^P-value	0.001	0.01	0.35	0.05	
**FBS (mg/dl)**	Before	161.68 ± 53.83	159.91 ± 55.59	170.61 ± 64.14	151.78 ± 41.33	0.69
	After	162.29 ± 64.96	162.68 ± 50.92	178.36 ± 69.14	153.05 ± 32.25	
	Mean change	-1.29 ± 37.68	2.77 ± 22.80	7.27 ± 53.91	1.09 ± 38.12	
	^**^P-value	0.87	0.57	0.53	0.90	
**TC (mg/dl)**	Before	166.20 ± 41.61	176.14 ± 26.05	181.52 ± 26.05	161.83 ± 28.26	0.44
	After	158.67 ± 39.51	167.77 ± 27.98	184.18 ± 32.53	160.73 ± 32.07	
	Mean change	-8.87 ± 16.39	-8.36 ± 29.28	1.36 ± 18.29	-1.27 ± 19.26	
	^**^P-value	0.01	0.19	0.73	0.76	
**LDL-C (mg/dl)**	Before	82.64 ± 25.83	80.73 ± 27.27	88.96 ± 17.36	76.78 ± 17.48	0.89
	After	88.60 ± 26.66	88.04 ± 35.32	99.86 ± 21.39	85.95 ± 22.05	
	Mean change	6.87 ± 23.95	7.32 ± 21.36	10.09 ± 14.76	9.14 ± 14.28	
	^**^P-value	0.33	0.12	0.40	0.07	
**TG (mg/dl)**	Before	154.44 ± 50.01	170.50 ± 76.20	127.74 ± 56.45	129.26 ± 34.2	0.12
	After	117.67 ± 45.63	139.00 ± 73.89	123.68 ± 43.19	116.82 ± 43.30	
	Mean change	-40.20 ± 26.63	-31.50 ± 39.37	-7.63 ± 30.60	-9.95 ± 39.89	
	^**^P-value	< 0.001	0.001	0.25	0.25	
**HDL-C (mg/day)**	Before	44.28 ± 8.83	44.18 ± 6.22	48.65 ± 10.42	42.04 ± 8.80	0.94
	After	49.79 ± 9.90	47.68 ± 7.27	50.64 ± 9.61	45.18 ± 7.79	
	Mean change	5.50 ± 7.92	3.50 ± 7.33	1.95 ± 8.58	3.09 ± 6.76	
	^**^P-value	0.002	0.03	0.30	0.04	
**Insulin (μU/ml)**	Before	22.25 ± 17.82	16.05 ± 9.75	15.60 ± 11.71	15.09 ± 4.81	0.21
	After	10.85 ± 6.63	9.20 ± 4.26	14.21 ± 12.55	10.53 ± 5.89	
	Mean change	-11.31 ± 16.39	-6.85 ± 10.49	-1.61 ± 7.49	-4.38 ± 6.25	
	^**^P-value	0.003	0.006	0.32	0.003	
**HOMA-IR**	Before	4.58 ± 1.36	4.34 ± 1.30	4.61 ± 1.67	4.12 ± 1.05	0.54
	After	4.27 ± 1.65	4.24 ± 1.33	4.75 ± 1.75	4.04 ± 0.86	
	Mean change	-0.31 ± 1.06	-0.10 ± 0.50	0.14 ± 1.30	-0.81 ± 0.90	
	^**^P-value	0.17	0.36	0.63	0.70	

Abbreviations: *BMI* body mass index, *SBP* systolic blood pressure, *DBP* diastolic blood pressure, *FBS* fasting blood sugar, *TC* total cholesterol, *LDL-C* low-density lipoprotein cholesterol, *TG* triglycerides, *HDL-C* high-density lipoprotein cholesterol; HOMA-IR, homeostatic model assessment for insulin resistance

Values are expressed as means ± SD. *P* < 0.05 was considered as statistically significant

^*^Analysis of covariance (ANCOVA) was used for comparisons between the groups post-intervention after adjusting for baseline values plus other confounders such as age, sex, BMI, and physical activity

^a^significantly different to group 3

^**^Paired sample t-test was used for comparisons between the means before and after the interventions

*P* < 0.05 was considered statistically significant

Regarding between-group differences, the results of the post-hoc analyses showed that probiotics supplement significantly reduced SBP levels compared to conventional milk. (*p* < 0.05). There were no significant differences between the groups in terms of other parameters (*p*˃0.05).

## Discussion

This randomized double-blind clinical trial aimed to examine the effects of soymilk and probiotics supplement co-administration on cardiovascular risk factors in patients with T2DM. The results showed soymilk plus probiotics supplement consumption significantly improved DBP, TC, TG, HDL-C, and insulin levels after 6 weeks of treatment. Also, soymilk consumption had significant beneficial effects on DBP, TG, HDL-C, and insulin levels. Moreover, probiotics supplementation significantly improved the levels of SBP, HDL-C, and insulin. The results of between-group comparisons showed that probiotics supplement significantly reduced SBP levels compared to conventional milk. As far as we know the majority of previous studies focused on fermented soymilk with specific probiotics and no studies have been conducted on soymilk plus probiotics co-administration.

In this study, we failed to show a significant difference in the reduction of anthropometric measurements, blood pressure, lipid profiles, and glucose tolerance indices by soymilk between the study's groups. However, within-group comparison showed significant beneficial effects of soymilk on DBP, TG, HDL-C, and insulin levels. A systematic review and meta-analysis investigated the effects of soymilk consumption on cardiovascular risk factors and reported inconsistent findings. Accordingly, soymilk consumption significantly decreased SBP, DBP, TC, LDL-C, waist circumference (WC), C-reactive protein (CRP), and tumor necrosis factor-alpha compared to the control group. However, it was not effective in the reduction of other risk factors including body weight, BMI, HDL-C, TG, FBS, and fasting insulin. The results differed from ours because the authors did not take into account the health status of the subjects and pooled data from healthy and sick subjects in the analysis. Also, none of the primary studies included in the meta-analyses were performed on patients with T2DM [33]. In a randomized crossover trial of T2DM patients with nephropathy, it has been found that soymilk consumption had significant beneficial effects on SBP and DBP levels, but not other cardiovascular risk factors [34]. In contrast to our study, the authors didn’t control the analyses for baseline values and other possible confounders in that study and that study had a crossover design of 4 weeks. In another study, the researchers used soymilk as a control group for probiotic soymilk. In that RCT, the authors found a significant effect of soymilk consumption on BMI after 4 weeks of intervention [23]. As the authors reported in another article, soymilk consumption was not effective in the reduction of other cardiovascular risk factors [24]. Accordingly, since that study considered soymilk as a control group, whereas it was considered an intervention group in our study, it is illogical to compare these results with ours. Regarding other studies that have not exclusively focused on soymilk, the results of a meta-analysis study didn't show the positive effects of soy protein consumption on body weight and WC when compared to casein/whole-milk protein. Also, this study showed that soy isoflavones consumption had no significant positive effects on BMI in the women population [35]. In contrast to our results, a systematic review and meta-analysis of RCTs indicated possible significant positive effects of soy products on serum TG, TC, LDL-C, and CRP concentrations in patients with T2DM. In that study, soy consumption was not effective in the reduction of other cardiovascular risk factors. It should also be noted that these results come from pooling data for each type of soy product, but not exclusively for soymilk [19]. So, according to available evidence, there are no possible beneficial effects of soymilk on cardiovascular risk factors in patients with T2DM, and further RCTs with larger sample sizes, longer durations, different soymilk dosages, and considering a control group for soymilk are warranted.

In our study, probiotics supplementations significantly improved the levels of SBP, HDL-C, and insulin after 6 weeks of intervention. The results of between-group comparisons also showed that probiotics supplement significantly reduced SBP levels compared to conventional milk. In a systematic review and meta-analysis of 13 RCTs, the authors found that multi-strain probiotics supplementation compared to the control group significantly improved FBS, HOMA-IR, TC, TG, SBP, and DBP, but not HbA1c, fasting insulin, HDL-C, and LDL-C [36]. In contrast to our study, the control group for those primary studies was a placebo capsule but not conventional milk plus a placebo capsule. In addition, the findings from subgroup analyses showed that the results may affect by several parameters such as study duration, country of study conduction, age, and baseline BMI. Moreover, the significant results from that study don’t appear to be clinically meaningful. Accordingly, the possible beneficial effects of multi-strain probiotics supplementation on cardiovascular risk factors in patients with T2DM patients are not well confirmed based on the available evidence and should be considered in future research.

Finally, this study indicated that soymilk plus probiotics supplement consumption significantly improved DBP, TC, TG, HDL-C, and insulin levels after 6 weeks of treatment. However, between-group comparisons didn't show significant differences. Few RCTs have investigated the effects of fermented soymilk on cardiovascular risk factors [23, 24, 37, 38]. Accordingly, in a study, intervention with fermented soymilk (200 ml/day) with *L. Plantarum* A7 significantly improved SBP, DBP, HDL-C, and TG levels, but not serum levels of adiponectin, LDL-C, FBS, some oxidative stress factors, inflammatory markers, and anthropometric measurements compared to pure soymilk (200 ml/day) in patients with T2DM after 8 weeks [23, 24, 37]. In contrast to our study, that research was conducted on diabetic patients with kidney disease, had longer durations, and used single-strain probiotics. Another crossover study in participants at high risk for cardiovascular risk factors showed that 12 weeks of intervention with fermented soy powder versus sprouted brown rice powder significantly decreased TC, LDL-C, and HDL-C concentrations [38]. Unlike our study, in that study the study population was not diabetic patients, the design of the study was crossover, the intervention was soy powder but not soymilk, and the study had a longer duration. Given our findings and the inconsistencies in the literature regarding the lipid-lowering potential of soy foods, it is of further interest to explore what factors may have contributed to these nonsignificant results. Baseline parameters status, supplement type, supplement dosage, and duration of the study are all potential confounding factors that have been identified and are discussed below. Some studies suggest that initial plasma levels are an important factor, as hypocholesterolemic effects only occur in patients with TC values of > 270 mg/dl [39]. Another reason could be because of the type and the amount of soymilk provided. While according to evidence, about 20 g of soy protein per day can lower non-HDL cholesterol and have favorable effects on the cardiovascular system [40]. The amount of protein in 240 ml of soymilk per day in this study may have been insufficient to significantly improve blood lipid levels and other parameters. The other explanation for these null results is that the level of isoflavones in one cup per day of soymilk was not sufficient to significantly improve relevant cardiovascular risk factors. Regarding isoflavone content, a recent meta-analysis indicated that consumption of soy protein coupled with isoflavones above 40 mg/day has lipid-lowering effects. Although the isoflavone content of the soymilk used in our study was not analyzed, Gardner et al. used a similar product and showed that three cups of soymilk supply approximately 90 mg of isoflavones [41]. Furthermore, the absorption, metabolism, and bioavailability of isoflavones may be affected by gut microbiota, ethnic origin, gut transit time, fecal digestion rates, some dietary factors, and storage conditions of soymilk [42, 43]. Finally, these factors combined with the short duration of the study, the lower dose of the probiotics supplement, and the type of probiotics bacteria in the supplement might have a role in not seeing a significant effect in the majority of parameters in this study.

This study has some strengths. As far as we know this study for the first time has investigated the effects of soymilk plus probiotics supplement in patients with T2DM. Also, in our study, the effectiveness of the main group (soymilk plus probiotics supplement) was compared with a control (conventional milk) and its two components (soymilk and probiotics). Moreover, compliance with the study protocol was assessed during and at the end of the study, and major confounders were controlled for in the analyses. Like any other study, our study also has some limitations. First, our study seems to have a short duration which resulted in non-significant results. Second, the isoflavone content of the soymilks was not assessed. Third, we haven’t evaluated the survival rate of probiotics bacteria during passage through the gastrointestinal tract.

## Conclusions

Our study showed that consumption of soymilk plus probiotics supplement for 6 weeks didn't improve cardiovascular risk factors compared to the conventional milk, soymilk, and probiotics supplement. However, the results showed the beneficial effects of probiotics supplementation in lowering SBP compared to conventional milk. Accordingly, our results didn’t confirm the possible synergic effects of soy products along with probiotics bacteria in the improvement of cardiovascular risk factors in patients with T2DM. Therefore, due to the limitations of this study and the inconsistent results of the available evidence, studies with expanded settings are warranted.

## Data Availability

The data that support the findings of this study are available from the corresponding author upon reasonable request.
